# Supplementary material for: Changes in SGLT2i and GLP-1RA real-world initiator profiles following cardiovascular outcome trials: A Danish nationwide population-based study
Source: PLoS One. 2020 Mar 4;15(3):e0229621. doi: 10.1371/journal.pone.0229621 (PMC7055862; doi:10.1371/journal.pone.0229621)
Supplement: S1 File — (DOCX) [file pone.0229621.s001.docx]

Supplementary Appendix

**S1 table: Codes used in study**

**S2 fig: Quarterly number of initiators of individual glucose-lowering drugs in Denmark**

**S3 fig: Time trends in proportions using various baseline glucose-lowering drug regimens, at the time of initiation of new glucose-lowering drugs. Liraglutide 3.0 mg daily (obesity treatment label) included in the GLP-1RA analysis.**

**S4 table: Time trends in clinical characteristics from 2014-2017 among real-world initiators of SGLT2i**

**S5 fig: Time trends in proportions with established atherosclerotic cardiovascular disease (ASCVD) or hospital-diagnosed obesity at baseline among SGLT2i initiators**

**S6 table: Time trends in clinical characteristics from 2014-2017 among real-world initiators of GLP-1RA**

**S7 table: Time trends in clinical characteristics from 2014-2017 among real-world initiators of DPP-4i**

**S8 fig: Time trends in patient proportions treated with selected drugs at baseline**

| **S1 table: Codes used in study** | | |
| --- | --- | --- |
| **Variable** | **Operational definition in Danish databases based on prescription, hospital contact, and laboratory codes** | **Codes used in Danish databases International Statistical Classification of Diseases and Related Health Problems 10^th^ revision (ICD-10) and procedural codes** |
| **Age** | Age at time of first ever GLD |  |
| **HbA_1c_** **level** |  | All analysis codes for HbA1c tests in Labka research database(23) |
| **Diabetes duration** | Calendar time from initiation of first ever GLD to initiation of indexdrug | Drugs starting with ATC code “A10” |
| **Atherosclerotic cardiovascular disease (ASCVD)** | Ischemic Heart Disease,  Cerebrovascular disease,  Abdominal and peripheral vascular disease  All data 15 years before index date | "DI21" "DI23" "DI24" "DT822A" "DT823" "KFNA" "KFNB" "KFNC" "KFND" "KFNE" "KFNF" "KFNG" "KFNH" "KFNW" "KFLF" "DG45" "DI20" "DI25" "DG45" "DI672" "DI678" "DI679" "DI691" "DI693" "DI694" "DI695" "DI696" "DI697" "DI698" "DI708" "DI61" "DI63" "DI64" "DI65" "DI66” "KAAL10" "KAAL11" "KPAE" "KPAF" "KPAH" "KPAN" "KPAP" "KPAQ" "KPAW99" "KPAU74" "KPBE" "KPBF" "KPBH" "KPBN" "KPBP" "KPBQ" "KPBW" "KPGH10" "KPCE" "KPCF" "KPCH" "KPCN" "KPCP" "KPCQ" "KPCW99" "KPCW20" "KPCU74" "KPCU82" "KPCU83" "KPCU84" "KPGE" "KPGF" "KPGH" "KPGN" "KPGP" "KPGQ" "KPGW99" "KPGW20" "KPEE" "KPEF" "KPEH" "KPEN" "KPEP" "KPEQ" "KPEW" "KPFE" "KPFH" "KPFN" "KPFP" "KPFQ" "KPFW" "KPGH20" "KPGH21" "KPGH22" "KPGH23" "KPGH30" "KPGH31" "KPGH40" "KPGH99" "KPDU74" "KPDU82" "KPDU83" "KPDU84" "KPEU74" "KPEU82" "KPEU83" "KPEU84" "KPFU74" "KPFU82" "KPFU83" "KPFU84" "KPGU74" "KPGU83" "KPGU84" "KPGU99" "KPGW" "KPWG" "DI702" "DI742" "DI743" "DI744" "DI745" "DI739A" "DI739C" "DE105" "DE115" "DE145" "DI700" "DI739" "DI748" "DI749" "DI709" "DI740" "DI741" |
| **Cerebrovascular disease** | All data 15 years before index date | DI60-DI69 “DG45” “DG46” |
| **Peripheral vascular disease** | All data 15 years before index date | “DI70” “DI71” “DI72” “DI73” ”DI74” ”DI77” |
| **Diabetic retinopathy** | All data 15 years before index date | "DE103" "DE113" "DE143" "DH340" "DH341" "DH342" "DH280" "DH334" "DH450" "DH360" "DH540" "DH541" "DH544" "DH25" "DH268" "DH269" "DH430" "DH431" "DH438C" "DH439" "DH334A" "DH330" "DH335" "DH470"  "KCKC10" "KCKC15" "KCKD65" |
| **Diabetic nephropathy** | All data 15 years before index date | "DE102" "DE112" "DE142" "DI120" "DN083" "DN06" "DN17" "DN18" "DN19" "DR809"'BJFD2' |
| **Diabetic neuropathy** | All data 15 years before index date | "DE104" "DE114" "DE144" "DG590" "DG632" "DG598" "DG603" "DG628" "DG629" "DG632" "DG638" "DG990" |
| **Chronic heart failure** | All data 15 years before index date | “DI50” “DI110” “DI130” “DI132” |
| **Atrial fibrillation** | All data 15 years before index date | 'DI48' |
| **Hypertension** | All data 15 years before index date | 'DI10'-'DI15' |
| **COPD** | All data 15 years before index date | 'DJ40'-'DJ48''DJ60'-'DJ68'  'DJ684''DJ701''DJ703'  'DJ961''DJ982''DJ983' |
| **Cancer** | All data 15 years before index date | 'DC00'-'DC99' |
| **Renal disease** | All data 15 years before index date | 'DI12''DI13''DN00''DN06' 'DN07''DN11''DN14' 'DN17''DN20''DQ61' |
| **Rheumatic disease** | All data 15 years before index date | 'DM05''DM06''DM08''DM09'  'DM30''DM31''DM32''DM33' 'DM34''DM35''DD86' |
| **Osteoarthritis** | All data 15 years before index date | 'DM15'-'DM20' |
| **Osteoporosis/fracture** | All data 15 years before index date | 'DM80''DM83''DS72'-'DS722' 'DS724''DS526''DM485' |
| **History of infections requiring hospitalization** | All data 15 years before index date | DA00-DA09,DA15-DA44, DA46, DA48 -DA99, DB00-DB09, DB15-DB99, DE060, DE321, DG00-DG07, DH00, DH010, DH030, DH031, DH050, DH061, DH10, DH131, DH191, DH192, DH220, DH320, DH440, DH600, DH601, DH603, DH620, DH621, DH622, DH623, DH650, DH660-DH664, DH67, DH700, DH853, DI01, DI02, DI301, DI320, DI33, DI38, DI398, DI400, DJ00-DJ06, DJ10-D18, DJ20-22, DJ34, DJ36, DJ390, DJ391, DJ440, DJ851, DJ86,DK040, DK047, DK052, DK113, DK122, DK36, DK37, DK570, DK572, DK574, DK578, DK61, DK619, DK630, DK650, DK659, DK67, DK750, DK751, DK800, DK803, DK804, DK810, DK819, DK830, DK859, DL00-DL08, DM00, DM01, DM86, DM 631, DM632, DN10, DN12, DN151, DN30, DN330, DN340, DN341, DN390, DN41, DN45, DN70-DN77, DO23, DO264, DO411, DO740, DO753, DO85, DO86, DO883, DO91, DO98, DT802, DT814, DT826, DT827, DT835, DT836, DT845-DT847, DT857, DT880, DT899 |
| **Obesity** | All data 15 years before index date | 'DE65'-'DE68' |
| **Alcoholism** | All data 15 years before index date | 'DG312''DG621''DG721' 'DI426''DK292''DK860' 'DK70''DR780''DT51' 'DZ714''DZ721' |
| **Mental Disorders** | All data 15 years before index date | 'DF00'-'DF99' + 'N05A' 'N05BA' 'N05CD' 'N05CF' 'N06A' |
| **Previous hypoglycaemia** | All data 15 years before index date | 'DE160'-'DE162''DE15' 'DT383A' |
| **Trombocyte aggregation prophylaxis** | All data 15 years before index date | "B01AC06" "N02BA01" "B01AC30" "B01AC07" "B01AC22" "B01AC04" "B01AC24" |
| **Statins** | All data 15 years before index date | "C10AA" "C10BA" "C10BX" |
| **ACE inhibitors** | All data 15 years before index date | "C09A" "C09B" |
| **ATII antagonists** | All data 15 years before index date | "C09C" "C09D" |
| **Any antihypertensive treatment** | All data 15 years before index date | “C02” “C03”, “C07” “C08” “C09” |
| **Oral steriods** | All data 15 years before index date | "H02AB" |
| **Myocardial Infarction** | All data before index date (“ever before”) | “DI21” “DI22” “DI23” |

**S2 fig: Quarterly number of initiators of individual glucose-lowering drugs in Denmark,
2014-2017**

DPP-4i: dipeptidyl peptidase-4 inhibitor; GLP-1RA: glucagon-like peptide-1 receptor agonists including liraglutide used for obesity treatment; SGLT2i: sodium-glucose cotransporter 2 inhibitors; TECOS: Sitagliption (DPP-4i) showed non-inferiority to placebo(34); Lira obesity label: Liraglutide 3 mg launched as treatment for obesity; EMPA-REG OUTCOME: empagliflozin showed CV and CV/all-cause mortality benefits (10); LEADER: liraglutide showed CV and CV/all-cause mortality benefits (12); CANVAS: canagliflozin showed CV benefits (11).

**S3 fig: Time trends in proportions using various baseline glucose-lowering drug regimens,** **at the time of initiation of new glucose-lowering drugs.
Liraglutide 3.0 mg daily (obesity treatment label) included in the GLP-1RA analysis.**

*
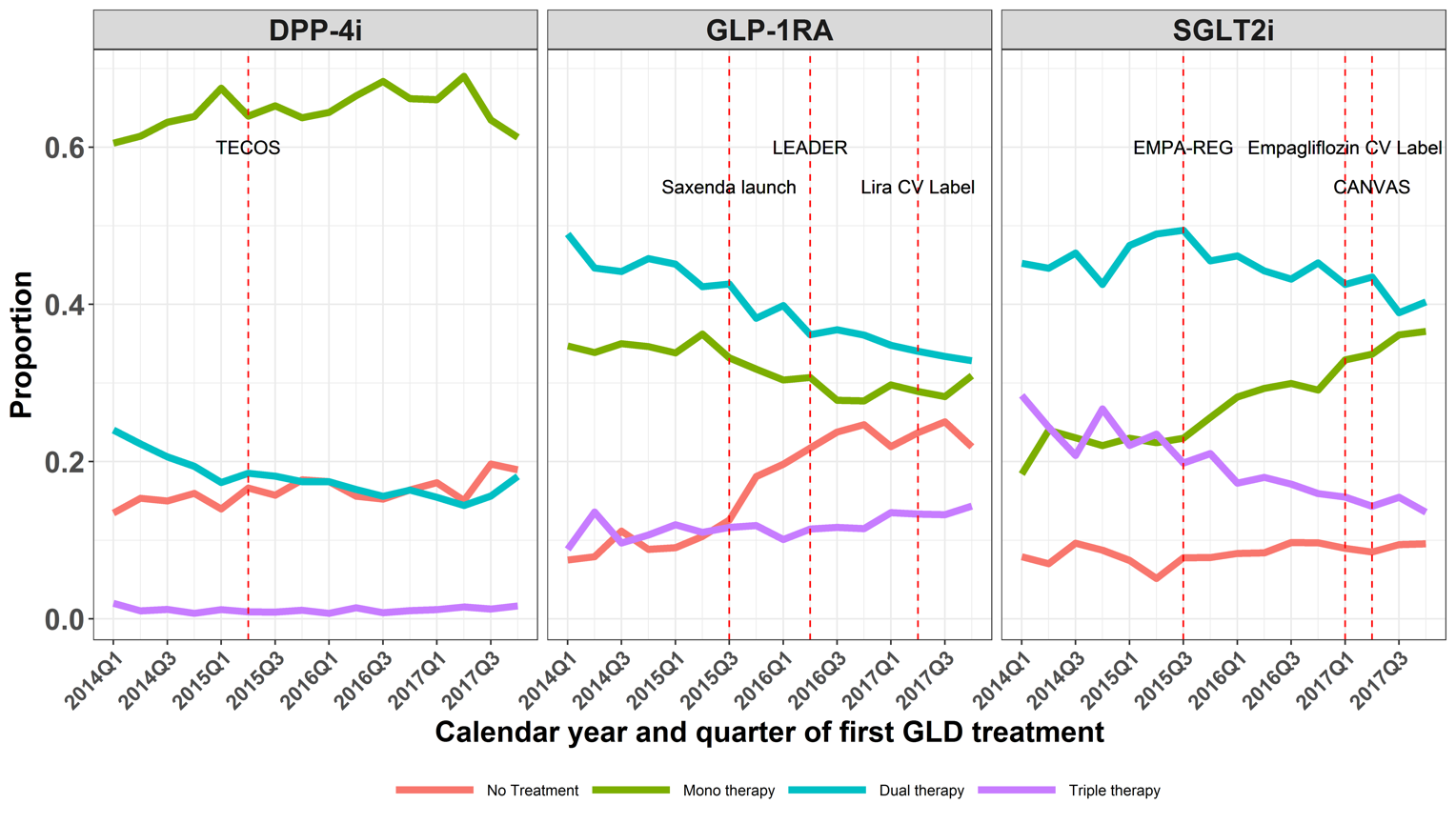
*

DPP-4i: dipeptidyl peptidase-4 inhibitor; GLP-1RA: glucagon-like peptide-1 receptor agonists ; SGLT2i: sodium-glucose cotransporter 2 inhibitors; TECOS: Sitagliption (DPP-4i) showed non-inferiority to placebo(34); Lira obesity label: Liraglutide 3 mg launched as treatment for obesity; EMPA-REG OUTCOME: empagliflozin showed CV and CV/all-cause mortality benefits (10); LEADER: liraglutide showed CV and CV/all-cause mortality benefits (12); CANVAS: canagliflozin showed CV benefits (11).

| **S4 table: Time trends in clinical characteristics from 2014-2017 among real-world initiators of SGLT2i** | | | | | | | | | |
| --- | --- | --- | --- | --- | --- | --- | --- | --- | --- |
|  | **2014** | | **2015** | | **2016** | | **2017** | | **2017 vs 2014 (ref.)** |
|  | **N** | **Percent (%)** | **N** | **Percent (%)** | **N** | **Percent (%)** | **N** | **Percent (%)** | **Prevalence Ratio**  **(adjusted for age and sex)** |
| **Sex** | 1193 | 39.6 | 1810 | 39.2 | 2862 | 37.9 | 3756 | 38.0 |  |
| Female |  |  |  |  |  |  |  |  | 0.96 (0.91-1.01) |
| Male | 1817 | 60.4 | 2803 | 60.8 | 4696 | 62.1 | 6133 | 62.0 | 1.03 (1.00-1.06) |
| **Age** |  |  |  |  |  |  |  |  |  |
| 0-29 | 17 | 0.6 | 22 | 0.5 | 47 | 0.6 | 76 | 0.8 | 1.37 (0.81-2.31) |
| 30-59 | 1422 | 47.2 | 2179 | 47.2 | 3457 | 45.7 | 4245 | 42.9 | 0.91 (0.87-0.95) |
| 60-69 | 1038 | 34.5 | 1483 | 32.1 | 2405 | 31.8 | 3116 | 31.5 | 0.91 (0.86-0.97) |
| 70+ | 533 | 17.7 | 929 | 20.1 | 1649 | 21.8 | 2452 | 24.8 | 1.40 (1.29-1.53) |
| **Region of residence** | 914 | 30.4 | 1462 | 31.7 | 2404 | 31.8 | 2926 | 29.6 |  |
| Capital Region |  |  |  |  |  |  |  |  | 0.97 (0.91-1.04) |
| Central Denmark Region | 723 | 24.0 | 1076 | 23.3 | 1605 | 21.2 | 2025 | 20.5 | 0.86 (0.79-0.92) |
| North Denmark Region | 215 | 7.1 | 400 | 8.7 | 680 | 9.0 | 1133 | 11.5 | 1.60 (1.39-1.84) |
| Region Zealand | 609 | 20.2 | 759 | 16.5 | 1313 | 17.4 | 1674 | 16.9 | 0.84 (0.77-0.91) |
| Southern Denmark | 549 | 18.2 | 916 | 19.9 | 1556 | 20.6 | 2131 | 21.5 | 1.18 (1.09-1.29) |
| **Diabetes duration** | 18 | 0.6 | 32 | 0.7 | 81 | 1.1 | 117 | 1.2 |  |
| 0 days |  |  |  |  |  |  |  |  | 2.01 (1.22-3.31) |
| 0-<2 year | 228 | 7.6 | 347 | 7.5 | 750 | 9.9 | 1215 | 12.3 | 1.67 (1.46-1.91) |
| 2-<5 years | 552 | 18.3 | 814 | 17.6 | 1231 | 16.3 | 1586 | 16.0 | 0.89 (0.82-0.97) |
| 5-<10 years | 1085 | 36.0 | 1688 | 36.6 | 2664 | 35.2 | 3304 | 33.4 | 0.93 (0.88-0.98) |
| 10+ years | 1127 | 37.4 | 1732 | 37.5 | 2832 | 37.5 | 3667 | 37.1 | 0.95 (0.90-1.00) |
| **ASCVD** | 827 | 27.5 | 1327 | 28.8 | 2153 | 28.5 | 2934 | 29.7 | 1.03 (0.97-1.10) |
| **Myocardial Infarction** | 583 | 19.4 | 958 | 20.8 | 1582 | 20.9 | 2205 | 22.3 | 1.10 (1.01-1.19) |
| **Cerebrovascular disease** | 231 | 7.7 | 343 | 7.4 | 566 | 7.5 | 831 | 8.4 | 1.04 (0.90-1.19) |
| **Abdominal and peripheral vascular disease** | 281 | 9.3 | 419 | 9.1 | 642 | 8.5 | 831 | 8.4 | 0.85 (0.75-0.97) |
| **Diabetic retinopathy** | 677 | 22.5 | 993 | 21.5 | 1522 | 20.1 | 1998 | 20.2 | 0.85 (0.79-0.92) |
| **Diabetic nephropathy** | 184 | 6.1 | 308 | 6.7 | 475 | 6.3 | 581 | 5.9 | 0.94 (0.80-1.10) |
| **Diabetic neuropathy** | 296 | 9.8 | 372 | 8.1 | 633 | 8.4 | 757 | 7.7 | 0.74 (0.66-0.85) |
| **Charlson comorbidty level (CCI)** † |  |  |  |  |  |  |  |  |  |
| Comorbidity level 0 | 1964 | 65.2 | 2960 | 64.2 | 4838 | 64.0 | 6147 | 62.2 | 0.97 (0.94-1.00) |
| Comorbidity level 1 | 584 | 19.4 | 894 | 19.4 | 1433 | 19.0 | 1891 | 19.1 | 0.97 (0.89-1.06) |
| Comorbidity level 2 | 286 | 9.5 | 463 | 10.0 | 772 | 10.2 | 1088 | 11.0 | 1.10 (0.97-1.25) |
| Comorbidity level >=3 | 176 | 5.8 | 296 | 6.4 | 515 | 6.8 | 763 | 7.7 | 1.22 (1.04-1.42) |
| **Chronic heart failure** | 152 | 5.0 | 230 | 5.0 | 428 | 5.7 | 656 | 6.6 | 1.22 (1.03-1.45) |
| **Atrial fibrillation** | 183 | 6.1 | 321 | 7.0 | 546 | 7.2 | 780 | 7.9 | 1.17 (1.00-1.36) |
| **Hypertension** | 1285 | 42.7 | 1877 | 40.7 | 3128 | 41.4 | 4023 | 40.7 | 0.92 (0.88-0.97) |
| **COPD** | 265 | 8.8 | 406 | 8.8 | 654 | 8.7 | 917 | 9.3 | 1.03 (0.91-1.18) |
| **Cancer** | 235 | 7.8 | 361 | 7.8 | 615 | 8.1 | 872 | 8.8 | 1.03 (0.90-1.19) |
| **Renal Disease** | 136 | 4.5 | 228 | 4.9 | 418 | 5.5 | 523 | 5.3 | 1.16 (0.97-1.39) |
| **Rheumatic disease** | 83 | 2.8 | 130 | 2.8 | 227 | 3.0 | 288 | 2.9 | 1.05 (0.83-1.34) |
| **Osteoarthritis** | 546 | 18.1 | 852 | 18.5 | 1397 | 18.5 | 1848 | 18.7 | 0.99 (0.91-1.08) |
| **Osteoporosis/fracture** | 36 | 1.2 | 61 | 1.3 | 98 | 1.3 | 137 | 1.4 | 1.08 (0.75-1.56) |
| **History of infections requiring hospitalization** | 996 | 33.1 | 1525 | 33.1 | 2504 | 33.1 | 3274 | 33.1 | 1.00 (0.94-1.06) |
| **Obesity** | 864 | 28.7 | 1232 | 26.7 | 1925 | 25.5 | 2344 | 23.7 | 0.85 (0.80-0.91) |
| **Alcoholism** | 32 | 1.1 | 46 | 1.0 | 75 | 1.0 | 80 | 0.8 | 0.76 (0.50-1.14) |
| **Mental Disorders** | 1663 | 55.2 | 2420 | 52.5 | 3911 | 51.7 | 5102 | 51.6 | 0.94 (0.90-0.97) |
| **Previous hypoglycaemia** | 35 | 1.2 | 61 | 1.3 | 63 | 0.8 | 102 | 1.0 | 0.84 (0.57-1.23) |
| **Trombocyte aggregation prophylaxis** | 1243 | 41.3 | 1905 | 41.3 | 2864 | 37.9 | 3618 | 36.6 | 0.85 (0.81-0.89) |
| **Statins** | 2332 | 77.5 | 3564 | 77.3 | 5805 | 76.8 | 7511 | 76.0 | 0.97 (0.95-0.99) |
| **ACE inhibitors** | 1250 | 41.5 | 1765 | 38.3 | 2909 | 38.5 | 3605 | 36.5 | 0.87 (0.82-0.91) |
| **ATII antagonists** | 1099 | 36.5 | 1651 | 35.8 | 2604 | 34.5 | 3427 | 34.7 | 0.93 (0.88-0.98) |
| **Any antihypertensive treatment** | 2469 | 82.0 | 3734 | 80.9 | 6051 | 80.1 | 7770 | 78.6 | 0.94 (0.92-0.96) |
| **Oral steriods** | 154 | 5.1 | 274 | 5.9 | 364 | 4.8 | 584 | 5.9 | 1.12 (0.94-1.33) |
| **Marital status** | 539 | 17.9 | 761 | 16.5 | 1250 | 16.5 | 1664 | 16.8 |  |
| Divorced |  |  |  |  |  |  |  |  | 1.22 (1.11-1.34) |
| Married | 1773 | 58.9 | 2670 | 57.9 | 4369 | 57.8 | 5652 | 57.2 | 0.99 (0.87-1.14) |
| Unknown | 46 | 1.5 | 56 | 1.2 | 50 | 0.7 | 45 | 0.5 | 0.94 (0.86-1.03) |
| Unmarried | 438 | 14.6 | 771 | 16.7 | 1279 | 16.9 | 1708 | 17.3 | 0.96 (0.93-1.00) |
| Widowed | 214 | 7.1 | 355 | 7.7 | 610 | 8.1 | 820 | 8.3 | 0.30 (0.20-0.46) |

**Abbreviations**: SGLT2i: Sodium-glucose co-transporter 2 inhibitors; aPR: adjusted Prevalence Ratio; ASCVD: atherosclerotic cardiovascular disease; COPD: Chronic obstructive pulmonary disease; ACE: angiotensin-converting-enzyme; ATII: angiotensin II receptor antagonists.

† Charlson Comorbity level calculated as a total of 0, 1, 2 or 3 and more.

**S5 fig: Time trends in proportions with established atherosclerotic cardiovascular disease (ASCVD) or hospital-diagnosed obesity at baseline among SGLT2i initiators**

Obesity: used hospital inpatient and outpatient contacts. EMPA-REG OUTCOME: empagliflozin showed CV and CV/all-cause mortality benefits [10], The figure is restricted to SGLT2i and stratified by individual drug types in order to address this question. We observe a slight increase since the time of the EMPAREG OUTCOME trial in the proportion with ASCVD for both dapagliflozin and empagliflozin initiators (canagliflozin starters are too few to yield statistically reliable trends). The ASCVD proportion was about 5 percentage points higher in empagliflozin than in dapagliflozin starters. This ASCVD difference between the 2 drugs was rather constant over time, and the difference was visible already before the EMPAREG outcome trial and not clearly affected by the trial publication.

| **S6 table: Time trends in clinical characteristics from 2014-2017 among real-world initiators of GLP-1RA** | | | | | | | | | |
| --- | --- | --- | --- | --- | --- | --- | --- | --- | --- |
|  | **2014** | | **2015** | | **2016** | | **2017** | | **2017 vs 2014 (ref.)** |
|  | **N** | **Percent (%)** | **N** | **Percent (%)** | **N** | **Percent (%)** | **N** | **Percent (%)** | **Prevalence Ratio**  **(adjusted for age and sex)** |
| **Sex** | 1282 | 44.0 | 1494 | 42.8 | 1679 | 44.5 | 1980 | 45.2 |  |
| Female |  |  |  |  |  |  |  |  | 1.03 (0.98-1.09) |
| Male | 1634 | 56.0 | 2000 | 57.2 | 2097 | 55.5 | 2405 | 54.8 | 0.97 (0.93-1.02) |
| **Age** |  |  |  |  |  |  |  |  |  |
| 0-29 | 47 | 1.6 | 55 | 1.6 | 79 | 2.1 | 89 | 2.0 | 1.25 (0.88-1.77) |
| 30-59 | 1490 | 51.1 | 1723 | 49.3 | 1869 | 49.5 | 2075 | 47.3 | 0.93 (0.88-0.97) |
| 60-69 | 900 | 30.9 | 1083 | 31.0 | 1097 | 29.1 | 1271 | 29.0 | 0.94 (0.88-1.01) |
| 70+ | 479 | 16.4 | 633 | 18.1 | 731 | 19.4 | 950 | 21.7 | 1.32 (1.19-1.46) |
| **Region of residence** | 915 | 31.4 | 1087 | 31.1 | 1023 | 27.1 | 1135 | 25.9 |  |
| Capital Region |  |  |  |  |  |  |  |  | 0.83 (0.77-0.89) |
| Central Denmark Region | 590 | 20.2 | 724 | 20.7 | 843 | 22.3 | 1061 | 24.2 | 1.20 (1.09-1.31) |
| North Denmark Region | 275 | 9.4 | 328 | 9.4 | 396 | 10.5 | 434 | 9.9 | 1.05 (0.91-1.21) |
| Region Zealand | 499 | 17.1 | 580 | 16.6 | 690 | 18.3 | 743 | 16.9 | 0.98 (0.89-1.09) |
| Southern Denmark | 637 | 21.8 | 775 | 22.2 | 824 | 21.8 | 1012 | 23.1 | 1.06 (0.97-1.15) |
| **Diabetes duration** | 71 | 2.4 | 83 | 2.4 | 140 | 3.7 | 148 | 3.4 |  |
| 0 days |  |  |  |  |  |  |  |  | 1.43 (1.09-1.89) |
| 0-<2 year | 400 | 13.7 | 496 | 14.2 | 533 | 14.1 | 596 | 13.6 | 1.03 (0.92-1.15) |
| 2-<5 years | 644 | 22.1 | 817 | 23.4 | 744 | 19.7 | 763 | 17.4 | 0.81 (0.73-0.88) |
| 5-<10 years | 898 | 30.8 | 1104 | 31.6 | 1223 | 32.4 | 1506 | 34.3 | 1.11 (1.04-1.19) |
| 10+ years | 903 | 31.0 | 994 | 28.4 | 1136 | 30.1 | 1372 | 31.3 | 0.95 (0.89-1.02) |
| **ASCVD** | 839 | 28.8 | 1018 | 29.1 | 1038 | 27.5 | 1190 | 27.1 | 0.90 (0.84-0.97) |
| **Myocardial Infarction** | 621 | 21.3 | 754 | 21.6 | 736 | 19.5 | 878 | 20.0 | 0.90 (0.82-0.98) |
| **Cerebrovascular disease** | 217 | 7.4 | 293 | 8.4 | 280 | 7.4 | 320 | 7.3 | 0.92 (0.78-1.09) |
| **Abdominal and peripheral vascular disease** | 284 | 9.7 | 294 | 8.4 | 331 | 8.8 | 336 | 7.7 | 0.75 (0.64-0.87) |
| **Diabetic retinopathy** | 563 | 19.3 | 633 | 18.1 | 680 | 18.0 | 764 | 17.4 | 0.84 (0.77-0.93) |
| **Diabetic nephropathy** | 189 | 6.5 | 276 | 7.9 | 290 | 7.7 | 342 | 7.8 | 1.14 (0.96-1.35) |
| **Diabetic neuropathy** | 257 | 8.8 | 257 | 7.4 | 253 | 6.7 | 293 | 6.7 | 0.73 (0.62-0.85) |
| **Charlson Comorbidity level (CCI)** † |  |  |  |  |  |  |  |  |  |
| Comorbidity level 0 | 1845 | 63.3 | 2171 | 62.1 | 2323 | 61.5 | 2685 | 61.2 | 0.98 (0.95-1.02) |
| Comorbidity level 1 | 546 | 18.7 | 693 | 19.8 | 739 | 19.6 | 826 | 18.8 | 0.99 (0.90-1.10) |
| Comorbidity level 2 | 319 | 10.9 | 387 | 11.1 | 405 | 10.7 | 492 | 11.2 | 0.98 (0.86-1.12) |
| Comorbidity level >=3 | 206 | 7.1 | 243 | 7.0 | 309 | 8.2 | 382 | 8.7 | 1.12 (0.96-1.32) |
| **Chronic heart failure** | 188 | 6.4 | 209 | 6.0 | 239 | 6.3 | 278 | 6.3 | 0.92 (0.77-1.09) |
| **Atrial fibrillation** | 196 | 6.7 | 272 | 7.8 | 292 | 7.7 | 353 | 8.1 | 1.07 (0.91-1.26) |
| **Hypertension** | 1213 | 41.6 | 1497 | 42.8 | 1549 | 41.0 | 1785 | 40.7 | 0.94 (0.89-0.99) |
| **COPD** | 294 | 10.1 | 338 | 9.7 | 407 | 10.8 | 472 | 10.8 | 1.04 (0.91-1.20) |
| **Cancer** | 224 | 7.7 | 259 | 7.4 | 336 | 8.9 | 389 | 8.9 | 1.05 (0.90-1.23) |
| **Renal Disease** | 167 | 5.7 | 174 | 5.0 | 219 | 5.8 | 272 | 6.2 | 1.07 (0.89-1.29) |
| **Rheumatic disease** | 93 | 3.2 | 111 | 3.2 | 145 | 3.8 | 169 | 3.9 | 1.16 (0.91-1.49) |
| **Osteoarthritis** | 523 | 17.9 | 652 | 18.7 | 759 | 20.1 | 901 | 20.5 | 1.09 (0.99-1.19) |
| **Osteoporosis/fracture** | 44 | 1.5 | 43 | 1.2 | 45 | 1.2 | 71 | 1.6 | 0.94 (0.65-1.37) |
| **History of infections requiring hospitalization** | 1070 | 36.7 | 1265 | 36.2 | 1385 | 36.7 | 1658 | 37.8 | 1.03 (0.97-1.09) |
| **Obesity** | 900 | 30.9 | 1069 | 30.6 | 1150 | 30.5 | 1349 | 30.8 | 1.01 (0.94-1.08) |
| **Alcoholism** | 37 | 1.3 | 40 | 1.1 | 37 | 1.0 | 41 | 0.9 | 0.73 (0.47-1.13) |
| **Mental Disorders** | 1629 | 55.9 | 1919 | 54.9 | 2001 | 53.0 | 2424 | 55.3 | 0.99 (0.95-1.03) |
| **Previous hypoglycaemia** | 38 | 1.3 | 35 | 1.0 | 29 | 0.8 | 61 | 1.4 | 1.02 (0.68-1.52) |
| **Trombocyte aggregation prophylaxis** | 1119 | 38.4 | 1292 | 37.0 | 1244 | 32.9 | 1436 | 32.7 | 0.81 (0.77-0.86) |
| **Statins** | 2190 | 75.1 | 2584 | 74.0 | 2702 | 71.6 | 3196 | 72.9 | 0.96 (0.93-0.98) |
| **ACE inhibitors** | 1185 | 40.6 | 1329 | 38.0 | 1388 | 36.8 | 1523 | 34.7 | 0.84 (0.80-0.89) |
| **ATII antagonists** | 930 | 31.9 | 1204 | 34.5 | 1252 | 33.2 | 1521 | 34.7 | 1.06 (0.99-1.13) |
| **Any antihypertensive treatment** | 2305 | 79.0 | 2792 | 79.9 | 2929 | 77.6 | 3432 | 78.3 | 0.97 (0.95-0.99) |
| **Oral steriods** | 160 | 5.5 | 217 | 6.2 | 229 | 6.1 | 303 | 6.9 | 1.21 (1.01-1.46) |
| **Marital status** | 491 | 16.8 | 582 | 16.7 | 683 | 18.1 | 763 | 17.4 |  |
| Divorced |  |  |  |  |  |  |  |  | 1.25 (1.14-1.37) |
| Married | 1661 | 57.0 | 1950 | 55.8 | 2029 | 53.7 | 2379 | 54.3 | 0.89 (0.76-1.03) |
| Unknown | 60 | 2.1 | 54 | 1.5 | 40 | 1.1 | 24 | 0.5 | 1.02 (0.92-1.14) |
| Unmarried | 487 | 16.7 | 631 | 18.1 | 743 | 19.7 | 877 | 20.0 | 0.94 (0.91-0.98) |
| Widowed | 217 | 7.4 | 277 | 7.9 | 281 | 7.4 | 342 | 7.8 | 0.28 (0.17-0.44) |

**Abbreviations**: GLP-1RA: glucagon-like peptide-1 receptor agonists; aPR: adjusted Prevalence Ratio; ASCVD: atherosclerotic cardiovascular disease; COPD: Chronic obstructive pulmonary disease; ACE: angiotensin-converting-enzyme; ATII: angiotensin II receptor antagonists;

† Charlson Comorbity level calculated as a total of 0, 1, 2 or 3 and more.

| **S7 table: Time trends in clinical characteristics from 2014-2017 among real-world initiators of DPP-4i** | | | | | | | | | |
| --- | --- | --- | --- | --- | --- | --- | --- | --- | --- |
|  | **2014** | | **2015** | | **2016** | | **2017** | | **2017 vs 2014 (ref.)** |
|  | **N** | **Percent (%)** | **N** | **Percent (%)** | **N** | **Percent (%)** | **N** | **Percent (%)** | **Prevalence Ratio**  **(adjusted for age and sex)** |
| **Sex** | 3180 | 39.6 | 3434 | 39.8 | 3544 | 40.0 | 3470 | 40.5 |  |
| Female |  |  |  |  |  |  |  |  | 1.02 (0.98-1.06) |
| Male | 4855 | 60.4 | 5191 | 60.2 | 5310 | 60.0 | 5095 | 59.5 | 0.99 (0.96-1.01) |
| **Age** |  |  |  |  |  |  |  |  |  |
| 0-29 | 40 | 0.5 | 44 | 0.5 | 54 | 0.6 | 60 | 0.7 | 1.40 (0.94-2.08) |
| 30-59 | 2774 | 34.5 | 3056 | 35.4 | 2949 | 33.3 | 3009 | 35.1 | 1.02 (0.98-1.06) |
| 60-69 | 2436 | 30.3 | 2504 | 29.0 | 2459 | 27.8 | 2212 | 25.8 | 0.85 (0.81-0.90) |
| 70+ | 2785 | 34.7 | 3021 | 35.0 | 3392 | 38.3 | 3284 | 38.3 | 1.10 (1.06-1.15) |
| **Region of residence** | 2469 | 30.7 | 2602 | 30.2 | 2528 | 28.6 | 2616 | 30.5 |  |
| Capital Region |  |  |  |  |  |  |  |  | 0.99 (0.95-1.04) |
| Central Denmark Region | 1857 | 23.1 | 1876 | 21.8 | 1938 | 21.9 | 1878 | 21.9 | 0.95 (0.90-1.01) |
| North Denmark Region | 897 | 11.2 | 1057 | 12.3 | 1013 | 11.4 | 922 | 10.8 | 0.96 (0.88-1.05) |
| Region Zealand | 1166 | 14.5 | 1243 | 14.4 | 1466 | 16.6 | 1336 | 15.6 | 1.07 (1.00-1.16) |
| Southern Denmark | 1646 | 20.5 | 1847 | 21.4 | 1909 | 21.6 | 1813 | 21.2 | 1.03 (0.97-1.10) |
| **Diabetes duration** | 301 | 3.7 | 370 | 4.3 | 340 | 3.8 | 364 | 4.2 |  |
| 0 days |  |  |  |  |  |  |  |  | 1.13 (0.97-1.31) |
| 0-<2 year | 1592 | 19.8 | 1728 | 20.0 | 1739 | 19.6 | 1831 | 21.4 | 1.09 (1.02-1.15) |
| 2-<5 years | 1958 | 24.4 | 2135 | 24.8 | 2002 | 22.6 | 1750 | 20.4 | 0.84 (0.80-0.89) |
| 5-<10 years | 2235 | 27.8 | 2429 | 28.2 | 2722 | 30.7 | 2577 | 30.1 | 1.08 (1.03-1.13) |
| 10+ years | 1949 | 24.3 | 1963 | 22.8 | 2051 | 23.2 | 2043 | 23.9 | 0.96 (0.91-1.02) |
| **Any macrovascular complication** | 2528 | 31.5 | 2626 | 30.4 | 2711 | 30.6 | 2505 | 29.2 | 0.91 (0.88-0.96) |
| **Myocardial Infarction** | 1843 | 22.9 | 1852 | 21.5 | 1960 | 22.1 | 1770 | 20.7 | 0.89 (0.84-0.94) |
| **Cerebrovascular disease** | 862 | 10.7 | 922 | 10.7 | 918 | 10.4 | 888 | 10.4 | 0.94 (0.86-1.03) |
| **Abdominal and peripheral vascular disease** | 758 | 9.4 | 724 | 8.4 | 705 | 8.0 | 654 | 7.6 | 0.79 (0.72-0.87) |
| **Diabetic retinopathy** | 1634 | 20.3 | 1722 | 20.0 | 1767 | 20.0 | 1725 | 20.1 | 0.96 (0.90-1.01) |
| **Diabetic nephropathy** | 722 | 9.0 | 795 | 9.2 | 843 | 9.5 | 791 | 9.2 | 1.01 (0.91-1.11) |
| **Diabetic neuropathy** | 500 | 6.2 | 465 | 5.4 | 480 | 5.4 | 436 | 5.1 | 0.80 (0.71-0.91) |
| **Charlson Comorbidity level (CCI)** † |  |  |  |  |  |  |  |  |  |
| Comorbidity level 0 | 4585 | 57.1 | 4914 | 57.0 | 4942 | 55.8 | 4831 | 56.4 | 0.99 (0.97-1.02) |
| Comorbidity level 1 | 1491 | 18.6 | 1584 | 18.4 | 1667 | 18.8 | 1521 | 17.8 | 0.95 (0.89-1.02) |
| Comorbidity level 2 | 1011 | 12.6 | 1082 | 12.5 | 1149 | 13.0 | 1076 | 12.6 | 0.98 (0.90-1.06) |
| Comorbidity level >=3 | 948 | 11.8 | 1045 | 12.1 | 1096 | 12.4 | 1137 | 13.3 | 1.09 (1.01-1.18) |
| **Chronic heart failure** | 652 | 8.1 | 694 | 8.0 | 741 | 8.4 | 681 | 8.0 | 0.95 (0.86-1.05) |
| **Atrial fibrillation** | 824 | 10.3 | 909 | 10.5 | 1028 | 11.6 | 985 | 11.5 | 1.07 (0.99-1.17) |
| **Hypertension** | 3205 | 39.9 | 3331 | 38.6 | 3500 | 39.5 | 3349 | 39.1 | 0.96 (0.93-1.00) |
| **COPD** | 799 | 9.9 | 884 | 10.2 | 951 | 10.7 | 917 | 10.7 | 1.06 (0.97-1.16) |
| **Cancer** | 883 | 11.0 | 963 | 11.2 | 1051 | 11.9 | 1067 | 12.5 | 1.10 (1.01-1.19) |
| **Renal Disease** | 485 | 6.0 | 509 | 5.9 | 599 | 6.8 | 583 | 6.8 | 1.13 (1.00-1.27) |
| **Rheumatic disease** | 291 | 3.6 | 312 | 3.6 | 328 | 3.7 | 329 | 3.8 | 1.03 (0.89-1.21) |
| **Osteoarthritis** | 1425 | 17.7 | 1565 | 18.1 | 1747 | 19.7 | 1698 | 19.8 | 1.10 (1.03-1.17) |
| **Osteoporosis/fracture** | 221 | 2.8 | 206 | 2.4 | 248 | 2.8 | 241 | 2.8 | 0.96 (0.81-1.15) |
| **History of infections requiring hospitalization** | 2871 | 35.7 | 3019 | 35.0 | 3137 | 35.4 | 3089 | 36.1 | 1.00 (0.96-1.05) |
| **Obesity** | 1278 | 15.9 | 1447 | 16.8 | 1489 | 16.8 | 1439 | 16.8 | 1.06 (0.99-1.13) |
| **Alcoholism** | 96 | 1.2 | 111 | 1.3 | 115 | 1.3 | 122 | 1.4 | 1.20 (0.92-1.56) |
| **Mental Disorders** | 4291 | 53.4 | 4537 | 52.6 | 4544 | 51.3 | 4395 | 51.3 | 0.96 (0.93-0.98) |
| **Previous hypoglycaemia** | 133 | 1.7 | 111 | 1.3 | 110 | 1.2 | 125 | 1.5 | 0.84 (0.66-1.06) |
| **Trombocyte aggregation prophylaxis** | 3303 | 41.1 | 3192 | 37.0 | 3050 | 34.4 | 2761 | 32.2 | 0.77 (0.74-0.80) |
| **Statins** | 5913 | 73.6 | 6222 | 72.1 | 6317 | 71.3 | 6014 | 70.2 | 0.95 (0.93-0.97) |
| **ACE inhibitors** | 3212 | 40.0 | 3289 | 38.1 | 3175 | 35.9 | 2900 | 33.9 | 0.85 (0.81-0.88) |
| **ATII antagonists** | 2388 | 29.7 | 2608 | 30.2 | 2752 | 31.1 | 2637 | 30.8 | 1.03 (0.98-1.08) |
| **Any antihypertensive treatment** | 6366 | 79.2 | 6760 | 78.4 | 6930 | 78.3 | 6573 | 76.7 | 0.96 (0.95-0.98) |
| **Oral steriods** | 628 | 7.8 | 685 | 7.9 | 711 | 8.0 | 689 | 8.0 | 1.01 (0.91-1.12) |
| **Marital status** | 1219 | 15.2 | 1334 | 15.5 | 1452 | 16.4 | 1456 | 17.0 |  |
| Divorced |  |  |  |  |  |  |  |  | 1.20 (1.12-1.29) |
| Married | 4575 | 56.9 | 4881 | 56.6 | 4919 | 55.6 | 4636 | 54.1 | 0.92 (0.86-0.99) |
| Unknown | 145 | 1.8 | 148 | 1.7 | 91 | 1.0 | 38 | 0.4 | 1.12 (1.05-1.20) |
| Unmarried | 1004 | 12.5 | 1129 | 13.1 | 1194 | 13.5 | 1277 | 14.9 | 0.95 (0.93-0.98) |
| Widowed | 1092 | 13.6 | 1133 | 13.1 | 1198 | 13.5 | 1158 | 13.5 | 0.25 (0.17-0.35) |

**Abbreviations**: DPP-4i: Dipeptidyl peptidase-4 inhibitor; aPR: adjusted Prevalence Ratio; ASCVD: atherosclerotic cardiovascular disease; COPD: Chronic obstructive pulmonary disease; ACE: angiotensin-converting-enzyme; ATII: angiotensin II receptor antagonists

† Charlson Comorbity level calculated as a total of 0, 1, 2 or 3 and more.

**S8 fig: Time trends in patient proportions treated with selected drugs at baseline**

DPP-4i: dipeptidyl peptidase-4 inhibitor; GLP-1RA: glucagon-like peptide-1 receptor agonists ; SGLT2i: sodium-glucose cotransporter 2 inhibitors; TECOS: Sitagliption (DPP-4i) showed non-inferiority to placebo(34); Lira obesity label: Liraglutide 3 mg launched as treatment for obesity; EMPA-REG OUTCOME: empagliflozin showed CV and CV/all-cause mortality benefits (10); LEADER: liraglutide showed CV and CV/all-cause mortality benefits (12); CANVAS: canagliflozin showed CV benefits (11).

**Abbreviations**: non-insulin GLD, non-insulin Glucose Lowering Drugs; eGFR, estimated glomerular filtration rate; ASCVD: atherosclerotic cardiovascular disease; COPD: Chronic obstructive pulmonary disease; ACE: angiotensin-converting-enzyme; ATII: angiotensin II receptor antagonists.
